# Differential Localization of Chemotactic Signaling Arrays during the Lifecycle of *Vibrio parahaemolyticus*

**DOI:** 10.3389/fmicb.2016.01767

**Published:** 2016-11-02

**Authors:** Jan Heering, Simon Ringgaard

**Affiliations:** Department of Ecophysiology, Max Planck Institute for Terrestrial MicrobiologyMarburg, Germany

**Keywords:** swarming, differentiation, chemotaxis, intracellular organization, ParC, *Vibrio parahaemolyticus*

## Abstract

When encountering new environments or changes to their external milieu, bacteria use elaborate mechanisms to respond accordingly. Here, we describe how *Vibrio parahaemolyticus* coordinates two such mechanisms – differentiation and chemotaxis. *V. parahaemolyticus* differentiates between two distinct cell types: short rod-shaped swimmer cells and highly elongated swarmer cells. We show that the intracellular organization of chemotactic signaling arrays changes according to the differentiation state. In swimmer cells chemotaxis arrays are strictly polarly localized, but in swarmer cells arrays form both at the cell poles and at irregular intervals along the entire cell length. Furthermore, the formation of lateral arrays increases with cell length of swarmer cells. Occurrence of lateral signaling arrays is not simply a consequence of the elongated state of swarmer cells, but is instead differentiation state-specific. Moreover, our data suggest that swarmer cells employ two distinct mechanisms for localization of polar and lateral signaling arrays, respectively. Furthermore, cells show a distinct differentiation and localization pattern of chemosensory arrays, depending on their location within swarm colonies, which likely allows for the organism to simultaneously swarm across surfaces while sustaining a pool of swimmers immediately capable of exploring new liquid surroundings.

## Introduction

**FIGURE 1:**
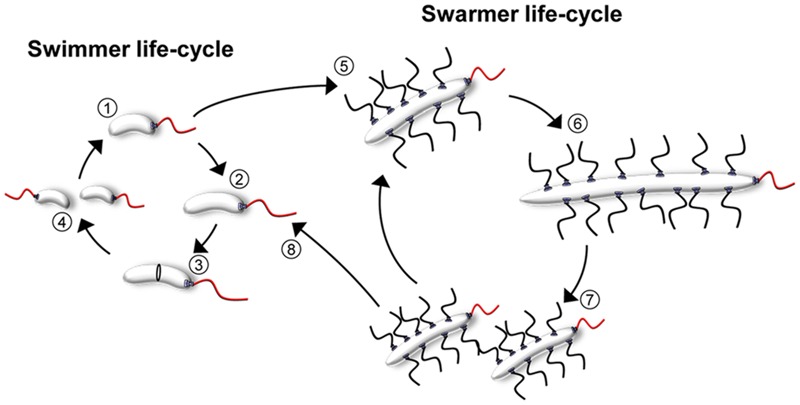
**The cell cycles of *V. parahaemolyticus*.** Schematic showing the life-styles and cell-cycles of *V. parahaemolyticus*. During the swimmer state, cells elongate (1–3) and eventually divide at mid-cell resulting in two progeny swimmer cells (4). Upon surface contact, swimmer cells can differentiate into the filamentous peritrichously flagellated swarmer cells (5). Swarmer cells can either continue the swarmer life-style, where division events result in swarmer progeny cells (5–7); alternatively swarmers can de-differentiate back into swimmer cells (8) and re-enter the swimmer cell cycle (1–4).

Bacteria often experience changes in their external environment and have developed various strategies to respond accordingly. One mechanism to accommodate such changes involves the differentiation into specialized cell types suitable for the particular conditions. Differentiation often involves major changes in the cell cycle, cell morphology, and the spatiotemporal organization of cells. A distinct type of differentiation utilized by many bacteria, including species of *Serratia* (Alberti and Harshey, 1990), *Aeromonas* (Kirov et al., 2002), *Salmonella* (Harshey, 1994; Harshey and Matsuyama, 1994), *Proteus* (Rather, 2005), and *Vibrio* (McCarter, 2004), is the differentiation between a planktonic swimmer cell and a swarmer cell that is specialized for movement over solid surfaces or in viscous environments (McCarter, 2004). One organism that undergoes such differentiation between swimmer and swarmer cells is *Vibrio parahaemolyticus*, a worldwide human pathogen and major cause of seafood related gastroenteritis (McCarter and Silverman, 1990; McCarter, 1999, 2004, 2010; Makino et al., 2003; Stewart and McCarter, 2003; Gode-Potratz and McCarter, 2011). In *V. parahaemolyticus* swimmer cells are short rod-shaped cells that – as the name suggests - are optimized for swimming in liquid environments. However, when they encounter a solid surface, differentiation into a swarmer cell is triggered. Swarmer cells exist within bacterial communities of swarm colonies where they spread over surfaces. Within swarm colonies, there are differences in cell size – and likely also cell-type – according to the position of cells within a swarm colony (Belas and Colwell, 1982; Roth et al., 2013). In the periphery of the swarm colony, cells assemble into flares that extend outward from the colony and cells stacked in a few layers. Closer to the center of the swarm colony cells are stacked in multiple layers and are considerably shorter than cells in the flares. Swarmer cells can maintain the swarmer lifestyle, where division events result in two new swarmer cells; alternatively, swarmers can de-differentiate back into swimmer cells, depending on the conditions (**Figure 1**). One of the first steps in swarmer differentiation is inhibition of cell division, resulting in highly elongated rod-shaped filamentous swarmer cells. A second major change during swarmer differentiation is the production of a multitude of lateral flagella, which are important for swarming behavior and likely used for surface contact, cell–cell contact, and interaction between groups of cells in order to coordinate their movement across surfaces (Baumann and Baumann, 1977; McCarter, 2004; Böttcher et al., 2016). Interestingly, the two flagellar systems used by swimmer and swarmer cells are distinct, but both appear to share the central chemotaxis system that is required for regulating chemotactic behavior and flagellar rotation (Sar et al., 1990).

Chemotaxis is an essential process for many motile bacteria to compete when encountering changes in their external milieu, and is one of the principal ways motile bacteria sense, respond, and adapt to changing environmental conditions. The process enables the bacteria to bias their movement away from unfavorable conditions and toward a more favorable external milieu (Wadhams and Armitage, 2004; Sourjik and Armitage, 2010). Chemotaxis is mediated by large multi-component clusters of signaling proteins, usually referred to as chemotactic signaling arrays. Chemotactic stimuli in the environment such as repellants or attractants are detected by chemosensory receptors termed “methyl-accepting chemotaxis proteins” (MCPs) at the cell surface. These receptors generally span the cytoplasmic membrane and interact in the cytoplasm with a histidine kinase, CheA. This interaction is stabilized by the cytoplasmic adaptor protein, CheW. If the signal perceived by the MCPs represents unfavorable environmental conditions, a phosphosignaling cascade via the histidine kinase CheA and the response regulator CheY is induced. Elevated levels of phosphorylated CheY increase the chance of a change in flagellar rotation and in consequence the direction of bacterial swimming – over time this results in a net movement toward more favorable conditions (Wadhams and Armitage, 2004; Sourjik and Armitage, 2010).

Chemotaxis has been extensively studied in the peritrichously flagellated bacterium *Escherichia coli*. In *E. coli*, array formation is believed to be a stochastic process (Thiem and Sourjik, 2008), resulting in localization of signaling arrays at the cell poles and non-regularly along the cell length (Sourjik and Berg, 2000). This pattern likely ensures that sensory arrays are localized in close proximity to the lateral flagella and that signaling arrays are stably inherited to the daughter cells at cell division. In other organisms, such as *Caulobacter crescentus, Pseudomonas aeruginosa, Rhodobacter sphaeroides*, and *V. parahaemolyticus*, chemosensory arrays are specifically localized at the cell poles (Alley et al., 1992; Maddock and Shapiro, 1993; Gestwicki et al., 2000; Wadhams et al., 2003; Bardy and Maddock, 2005). In particular, it was recently reported that chemotaxis arrays are exclusively directed to one or both cell poles in the polarly flagellated pathogens *V. cholerae* and *V. parahaemolyticus* by a novel mechanism (Ringgaard et al., 2011, 2014; Yamaichi et al., 2012). Here, the signaling arrays localize to the old flagellated cell pole immediately after cell division. Later in the cell cycle, the chemotaxis proteins are recruited to the new cell pole as the rod-shaped cell elongates, thus resulting in a bi-polar localization pattern; no lateral arrays are formed. The next cell division event then results in two daughter cells with one polar signal array each. It was recently discovered that proper polar localization and inheritance of signaling arrays depends on the ParA-like ATPase ParC (Ringgaard et al., 2011, 2014). In the absence of ParC, chemotaxis proteins are no longer recruited to the cell poles correctly. Instead, signaling arrays form and localize randomly along the cell length. As a consequence, bi-polar localization is not established prior to cell division and both daughter cells do not inherit a signaling array upon cell division. Mislocalization and unsuccessful segregation of signaling arrays to daughter cells result in altered motility and decreased chemotaxis (Ringgaard et al., 2011, 2014). Interestingly, fluorescence microscopy studies have suggested that changes occur in the localization of signaling arrays during differentiation of *V. parahaemolyticus* and that signaling arrays do not only localize to the cell poles in swarmer cells but also along the length of the cell (Gestwicki et al., 2000).

Here, we performed an in-depth analysis of the localization of chemotactic signaling arrays in *V. parahaemolyticus* during its differentiation cycle and within swarm colonies. In contrast to swimmer cells, signaling arrays are not exclusively localized to the cell poles in swarmer cells, but also form distinct clusters that localize along the cell length. Interestingly, we show that there is a correlation between swarmer cell length and the number of signaling arrays formed within the swarmer cell, where the number of lateral clusters formed increases with increased cell length. Moreover, lateral arrays do not localize in regular intervals along the cells length but are distributed irregularly along the entire length of the swarmer cell, and on average each cell halve hold the same number of arrays. Our data suggest that this localization pattern is not a consequence of cell elongation *per se*, but instead formation of lateral sensory clusters is specific to the differentiated state of swarmer cells.

## Materials and Methods

### Growth Conditions and Media

If not otherwise stated *E. coli* and *V. parahaemolyticus* were grown in LB media or on LB agar plates at 30°C or 37°C containing antibiotics in the following concentrations: streptomycin 200 μg/ml; kanamycin 50 μg/ml; ampicillin 100 μg/ml; chloramphenicol 20 μg/ml for *E. coli* and 5 μg/ml for *V. parahaemolyticus*. When needed, L-arabinose was added to a final concentration of 0.2% w/v.

### Strains and Plasmids

The wild-type strain of *V. parahaemolyticus* used was the clinical isolate RIMD 2210633 and all mutants are derivatives of this strain. Strains and plasmids used throughout this study are listed in **Table 1**. Primers are listed in **Table 2**. *E. coli* strain DH5αλpir was used for standard cloning and SM10λpir was used for transfer of plasmid DNA by conjugation from *E. coli* to *V. parahaemolyticus*. Deletion of genes in *V. parahaemolyticus* was performed using standard allele exchange techniques using derivatives of suicide vector pDM4 (Milton et al., 1996).

**Table 1:** Strains and plasmids list.

Strain name	Genotype	Reference
*Vibrio parahaemolyticus* RIMD 2210633	Clinical isolate	Makino et al., 2003
*V. parahaemolyticus* MZ01	RIMD 2210633 Δ*vp2227 parC*	Ringgaard et al., 2014
*V. parahaemolyticus* SR58	RIMD 2210633 Δ*vp2225 cheW*	Ringgaard et al., 2014
*V. parahaemolyticus* JH2	RIMD 2210633 Δ*vpa1548 lafA*	This work
*V. parahaemolyticus* JH5	RIMD 2210633 Δ*vpa1538 lafK*	This work
*Escherichia coli* SM10λpir	KmR, *thi-1, thr, leu, tonA, lacY, supE, recA::RP4-2-Tc::Mu, λpir*	
*E. coli* DH5αλpir	*supE44, ΔlacU169 (ΦlacZΔM15), recA1, endA1, hsdR17, thi-1, gyrA96, relA1, λpir*	

**Plasmid name**	**Relevant genotype/description**	**Reference**

pMZ03	*P*BAD::*yfp-vp2225 (cheW)*	Ringgaard et al., 2014
pDM4	Suicide vector for construction of deletion mutants; *sacBR*; *oriR6K*; *Cm^R^*	Milton et al., 1996
pJH002	Plasmid for deletion of *lafK*	This work
pJH003	Plasmid for deletion of *lafA*	This work

**Table 2:** Primers list.

Primer number	Primer sequence
63	tcgtcatcattgaaccttaaccttc
64	gaaggttaaggttcaatgatgacgacggtattgatttacagtcggct
68	ataaagccatcttagtctccttag
69	ctaaggagactaagatggctttatggcaatgtctctacttcgttaata
146	ccccctctagatctcgtcgatttgtattccgtaaag
147	ccccctctagaactctctaagaccgagacaatc
148	ccccctctagatgagcgtattgctgaatttgatcc
149	ccccctctagattatgtgttccgccttcctctc

### Construction of Plasmids

#### Plasmid pJH002

The up- and down-stream regions flanking *vpa1538* were amplified using primer pairs 146/63 and 64/147, respectively, using *V. parahaemolyticus* RIMD 2210633 chromosomal DNA as template. In a third PCR, using primers 146/147 and products of the first two PCR reactions as template, the flanking regions were stitched together. The resulting product was digested with XbaI and was inserted into the equivalent site of pDM4, resulting in plasmid pJH002.

#### Plasmid pJH003

The up- and down-stream regions flanking *vpa1548* were amplified using primer pairs 148/68 and 69/149, respectively, using *V. parahaemolyticus* RIMD 2210633 chromosomal DNA as template. In a third PCR, using primers 148/149 and products of the first two PCR reactions as template, the flanking regions were stitched together. The resulting product was digested with XbaI and was inserted into the equivalent site of pDM4, resulting in plasmid pJH003.

### Microscopy

Fluorescence microscopy of swarming *V. parahaemolyticus* cells was carried out in several steps; 5 mL LB supplemented with the required antibiotic was inoculated with a colony of cells harboring the relevant plasmid and grown to OD600 = 0.1 at 37°C and shaking. Expression of fluorescent fusion proteins was induced by adding L-arabinose to a final concentration of 0.2% w/v. The cultures were incubated for an additional 2 h before 1.5 μL were spotted in the center of a swarming agar plates, which subsequently were sealed with scotch-tape. Swarming agar plates were prepared from 40 g/L “Difco Heart Infusion Agar” (BD) supplemented with the required antibiotic, 4 mM CaCl^2^, 50 μM 2,2′-Bipyridyl (Sigma–Aldrich), and 0.2% L-arabinose. The sealed plates were then incubated for 16–18 h at 24°C to induce swarming. For microscopy, a piece of swarming agar containing cells was cut out from the plate and mounted onto agarose pads (1% agarose w/v, 20% v/v PBS, and 20% v/v LB) on microscope slides.

In order to acquire stereomicroscopy images of swarming colonies, swarming plates were prepared as described, sealed and incubated for 16–18 h at 24°C. Before acquiring the images, scotch tape and the lid of the swarming plate was removed. Stereomicroscopy was carried out using a Leica M205 FA Stereomicroscope.

Preparation of *V. parahaemolyticus* swimmer cells for fluorescence microscopy was carried out essentially as described (Ringgaard et al., 2015; Briegel et al., 2016). A volume of 10 mL of LB was inoculated with a bacterial colony of *V. parahaemolyticus* from an over-night LB agar plate grown at 37°C and relevant antibiotic. Cells were incubated for 1 h at 37°C and shaking after which 0.2% w/v L-arabinose was added in order to induce expression of YFP-CheW. Cells were then incubated for additionally 2 h and subsequently mounted on an agarose pad and imaged using fluorescence microscopy. For Aztreonam treatment, Aztreonam (Fluka) was added to a final concentration of 30 μg/mL after the 2 h of induction with 0.2% w/v L-arabinose. Cells were then incubated for additional 30 min, mounted on an agarose pad and imaged by fluorescence microscopy. Fluorescence microscopy was carried out using a Nikon eclipse Ti inverted Andor spinning-disk confocal microscope equipped with a 100× lens and an Andor Zyla sCMOS cooled camera.

### Microscopy Image Analysis

Before analysis, images generated by Nikon NIS-Elements AR were split up in single channels using Fiji/ImageJ 1.49j10 and each channel was saved as a separate tiff image. DIC and the corresponding fluorescent channel were loaded in MetaMorph Oﬄine (version 7.7.5.0, Molecular Devices) where manual image analysis was performed. An overlay of both channels was generated before cells were marked using the “Multi-line tool.” The regions were then transferred to the original fluorescent channel image to extract the distances of foci and their distribution in *V. parahaemolyticus* cells. Using the line-scan histogram, foci positions could be marked and copied to an Excel spreadsheet where further analysis and calculations were performed.

### Demographic Analysis of Microscopy Data

Demographic analysis was performed in several steps: first fluorescence intensity profiles of cells were measured in Fiji/ImageJ, version 1.49j10. Afterward the generated data was processed in R [version 3.0.1; (R Development Core Team, 2008)] with a script that sorts cells by length and normalizes the generated intensity profiles as an average of each cell’s fluorescence. In R the ggplot2 package [version 1.0.0; (Wickham, 2009)] was used to produce the demographics.

### Electroporation of Plasmid DNA into *V. parahaemolyticus*

Electrical-competent *V. parahaemolyticus* cells were produced by inoculating a single colony from a fresh agar plate in 200 ml of LB medium and incubation at 37°C until an OD_600_ of 1.0 was reached. The cells were transferred onto ice immediately and all further steps were performed on ice and in pre-cooled centrifuges. The cells were harvested at 4°C for 10 min at 3500 rpm. After that, the supernatant was poured off and the cell pellet was re-suspended in 25 ml of ice-cold 273 mM sucrose solution (pH 7.4, buffered with KOH). The cells were again harvested at 4°C for 10 min at 3500 rpm. This washing step was performed twice. Afterward the washed cell pellet was re-suspended in 400 μl of ice-cold 273 mM sucrose solution with the addition of 15% glycerol. For the transformation, a 70 μl aliquot of electrocompetent cells was mixed with 100–1000 ng of plasmid DNA and transferred into an ice-cold electroporation cuvette (Gene Pulser^®^ Cuvette) and electroporated using the “GenePulser X-Cell” by BioRad with the following calibration: 25 μF, 2400 V and 200 Ω. Afterward the cells were incubated shaking in 600 μl LB medium at 37°C for 1–3 h. Later, the cultures where plated on selective LB agar plates and incubated at 37°C overnight.

## Results

### The Intracellular Organization of Chemosensory Arrays Changes during Differentiation of *V. parahaemolyticus*

**FIGURE 2:**
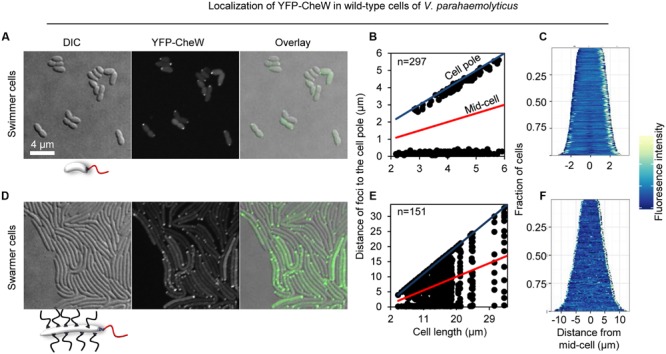
**Intracellular localization of chemotactic signaling arrays in swimmer and swarmer cells of *V. parahaemolyticus*.** Intracellular localization of YFP-CheW in wild-type swimmer **(A–C)** and swarmer cells **(D–F)** of *V. parahaemolyticus*. **(A,D)** Microscopy showing the intracellular localization of YFP-CheW in swimmer **(A)** and swarmer cells **(D)**. **(B,E)** Graph depicting the distance of YFP-CheW foci from the cell poles as a function of cell length in swimmer **(B)** and swarmer cells **(E)**. **(C,F)** demographic analysis showing the fluorescence intensity of YFP-CheW along the cell length in a population of *V. parahaemolyticus* relative to cell length in swimmer **(C)** and swarmer cells **(F)**.

**FIGURE 3:**
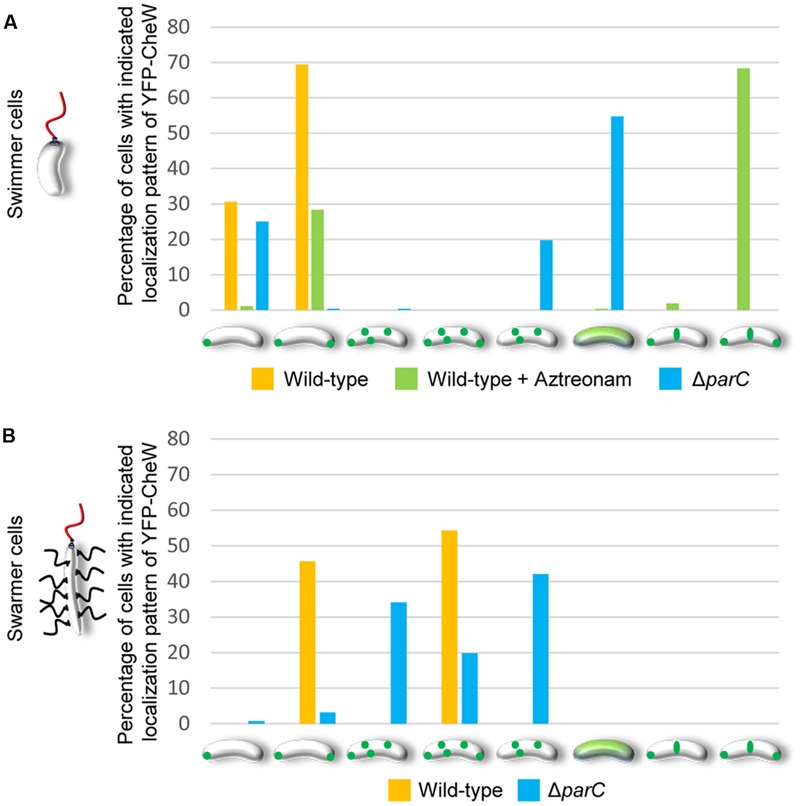
**Changes in the intracellular localization of chemotactic signaling arrays during differentiation of *V. parahaemolyticus*.** Localization pattern of YFP-CheW in swimmer **(A)** and swarmer cells **(B)** of wild-type, Aztreonam treated swimmer cells, and Δ*parC* strains of *V. parahaemolyticus*. Bar graphs show the percentage of cells with the indicated distinct localization patterns. **(A)** Number of cells analyzed, *n*: wild-type, *n* = 298; wild-type + Aztreonam, *n* = 272; Δ*parC, n* = 320. **(B)** Number of cells analyzed, *n*: wild-type, *n* = 151; Δ*parC, n* = 126.

In order to investigate the intracellular localization and organization of chemosensory arrays during differentiation of *V. parahaemolyticus*, we ectopically expressed a fluorescently tagged version of CheW (YFP-CheW) in swimmer and swarmer cells. Despite YFP-CheW not being able to fully complement a strain lacking CheW, we have previously shown it can be used as a marker for signaling array localization in presence of wild type CheW (Ringgaard et al., 2014). In swimmer cells, YFP-CheW localized in a uni- or bi-polar manner (**Figures 2A–C**) as previously reported: short cells displayed uni-polar localization whilst longer cells possessed bi-polar localization of signaling arrays (Ringgaard et al., 2014). Approximately 30% of cells had uni-polar localization whilst 70% of cells showed a bi-polar localization pattern (**Figure 3A**). Interestingly, the intracellular localization of signaling arrays was different for swarmer cells. In almost 100% of swarmer cells YFP-CheW was found at both cell poles (**Figure 3B**); importantly, the localization of YFP-CheW clusters was not restricted to cell poles. Instead, clusters of YFP-CheW could be observed localizing along the length of the cell (**Figures 2D–F**) in 55% of cells (**Figure 3B**). Thus, in contrast to swimmer cells, a large proportion of swarmer cells form and localize chemotactic signaling arrays along the length of the cell.

### Lateral Signaling Arrays are Distributed Through-Out the Length of the Cell in a Non-regular Manner

**FIGURE 4:**
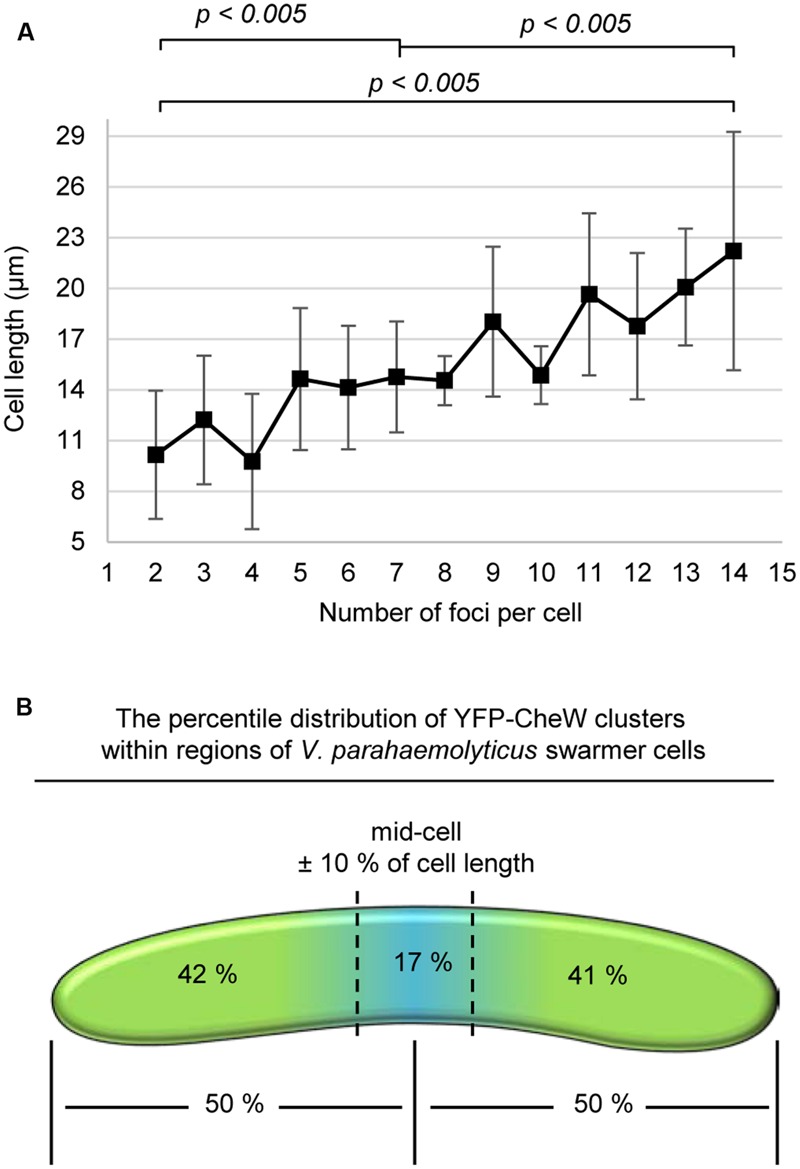
**A cell length dependent, non-regular distribution of lateral signaling arrays through-out the entire length of swarmer cells. (A)** Graph depicting the number of YFP-CheW clusters as a function of cell length. **(B)** Schematic showing the percentile distribution of YFP-CheW clusters within regions of *V. parahaemolyticus* swarmer cells.

In order to determine if there was a correlation between cell length and the number of lateral signaling arrays forming along the length of the cell in swarmer cells, we measured the number of YFP-CheW clusters as a function of cell length (**Figure 4A**). Interestingly, the number of lateral YFP-CheW clusters increased with increasing cell length. Thus, as swarmers elongate, there is a concomitant increase in the number of lateral signaling arrays.

Despite the non-regularity in array localization (**Figures 2E,F**), we analyzed if signaling arrays were distributed throughout the length of the cell. In order to do so, we calculated the percentile distribution of YFP-CheW clusters within specific regions of the cell (**Figure 4B**). On average each cell half possessed approximately 50% of signaling arrays, with 17% of arrays localizing within the mid-cell region (mid-cell ± 10% of cell length) (**Figure 4B**). Thus, signaling arrays are distributed along the entire length of the swarmer cell and on a population average each cell half contains the same number of signaling arrays.

### Lateral Localization of Signaling Arrays in Swarmer Cells Is Not a Function of Cell Length but Specific to the Differentiation State

**FIGURE 5:**
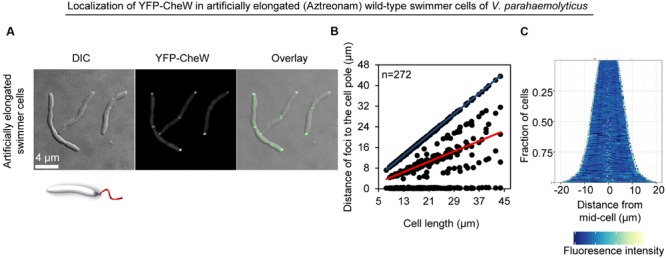
**The intracellular formation of chemotactic signaling arrays is cell type specific and not a consequence of swarm cell elongation.** Intracellular localization of YFP-CheW in wild-type swimmer cells of *V. parahaemolyticus* treated with the antibiotic Aztreonam in order to artificially induce elongation of swimmer cells. **(A)** Microscopy showing the intracellular localization of YFP-CheW. **(B)** Graph depicting the distance of YFP-CheW foci from the cell poles as a function of cell. **(C)** Demographic analysis showing the fluorescence intensity of YFP-CheW along the cell length in a population of *V. parahaemolyticus* cells relative to cell length.

We wanted to investigate if lateral signaling arrays also form in elongated cells from liquid media that have not initiated swarmer differentiation. Thus, we analyzed array localization in swimmer cells treated with the cell division inhibitor beta-lactam antibiotic Aztreonam. Aztreonam inhibits the function of the cell division protein FtsI, which results in the formation of elongated swimmer cells comparable in cell length to that of swarmer cells. Under these conditions, chemotactic signaling arrays were always localized at the cells poles (4% uni-polar and 96% bi-polar localization) and as regular bands corresponding to mid-cell or quarter-cell positions in 70% of cells (**Figures 3A** and **5**), suggesting an association of YFP-CheW with the cell division apparatus. This implies that lateral localization of signaling arrays along the cell length observed in swarmer cells is not simply a consequence of the elongated state of swarmer cells, but is in fact due to specific changes in the intracellular organization and localization of signaling arrays during differentiation between swimmer and swarmer cells.

### ParC Is Required for Polar Localization of Signaling Arrays in Swimmer and in Swarmer Cells

**FIGURE 6:**
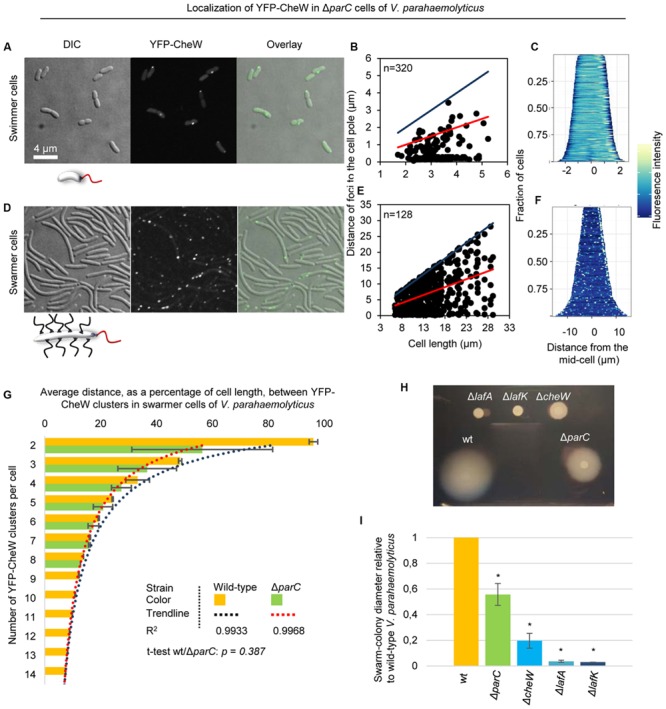
**Localization of chemotactic signaling arrays in the absence of ParC.** Intracellular localization of YFP-CheW in *V. parahaemolyticus*Δ*parC* swimmer **(A–C)** and swarmer cells **(D–F)**. **(A,D)** Microscopy showing the intracellular localization of YFP-CheW in swimmer **(A)** and swarmer cells **(D)** deleted for *parC*. **(B,E)** Graph depicting the distance of YFP-CheW foci from the cell poles as a function of cell length in swimmer **(B)** and swarmer cells **(E)** deleted for *parC*. **(C,F)** demographic analysis showing the fluorescence intensity of YFP-CheW along the cell length in a population of *V. parahaemolyticus* relative to cell length in swimmer **(C)** and swarmer cells **(F)** deleted for *parC*. **(G)** Bar graph showing the average distance between YFP-CheW clusters relative to cell length as a function of the number of YFP-CheW clusters per cell in wild-type and Δ*parC* swarmer cells of *V. parahaemolyticus*. Data for wild-type is shown in orange and Δ*parC* in green. The *R*^2^ value refer to the significance of the trendline. The *t*-test refer to the similarity between wild-type and a Δ*parC* strain. **(H)** Swarming colony formation of wild-type (wt), Δ*lafA*, Δ*lafK*, Δ*cheW*, and Δ*parC* strains of *V. parahaemolyticus*. **(I)** Diameter of swarm-colonies of wild-type (wt), Δ*lafA*, Δ*lafK*, Δ*cheW*, and Δ*parC* strains of *V. parahaemolyticus* relative to wild-type. Asterisk indicates *p < 0.005*.

We have previously shown that the ParC-system is responsible for polar localization of signaling arrays in swimmer cells. In order to determine if ParC also directs localization of signaling arrays in swarmer cells, we analyzed the localization in YFP-CheW in a strain deleted for *parC* and compared it to wild-type. As expected, signaling arrays were no longer recruited to the cell poles in the absence of ParC in swimmer cells, but formed clusters at random positions along the cell length. Consequently daughter cells did not faithfully inherit an array at cell division (**Figures 3A** and **6A–C**) (Ringgaard et al., 2011, 2014). Strikingly, in swarmer cells lacking ParC (**Figures 6D–F**) merely 23% of cells showed a bi-polar localization pattern of YFP-CheW (**Figure 3B**). Instead, YFP-CheW was localized uni-polarly in 45% of cells and 33% of cells completely lacked polar YFP-CheW clusters (**Figure 3B**). Thus, approximately 77% of swarmer cells failed to establish bi-polar localization in the absence of ParC (**Figures 3B** and **6D–F**). These data show that ParC is required for polar recruitment of YFP-CheW and chemotactic signaling arrays to the cell poles in both swimmer and swarmer cells.

### Formation and Localization of Lateral Signaling Arrays in Swarmer Cells Is Independent of ParC

Interestingly, chemotactic sensory arrays still formed and localized along the length of the cell in swarmer cells in absence of ParC. In order to analyze if the distribution of lateral signaling arrays was dependent on ParC, we measured the average distance between adjacent clusters of YFP-CheW relative to cell length and the number of YFP-CheW clusters per cell in wild-type and Δ*parC* backgrounds (**Figure 6G**). As expected the average distance between clusters of YFP-CheW decreased as the number of clusters increased. Interestingly, the average distance between clusters was indistinguishably between wild-type and cells lacking ParC (*p = 0.387*) (**Figure 6G**), hence indicating that localization and spacing between lateral signaling arrays is independent of the ParC-system and is guided by a different mechanism.

### ParC Is Required for Optimal Swarming Behavior

Since ParC is required for establishing bi-polar localization of chemotactic signaling arrays in swarmer cells, we investigated if ParC was required for swarming behavior of *V. parahaemolyticus*. To this end, we performed swarming assays with strains lacking ParC and compared it to wild-type cells. As negative controls for swarming, we included strains lacking the major lateral flagellin LafA or the swarmer specific sigma-factor LafK. Additionally, we included a strain lacking the chemotaxis protein CheW that does not show chemotactic behavior of swimmer cells (Ringgaard et al., 2014). As expected, wild-type cells formed large swarm colonies when spotted on swarm-agar plates and no swarming was observed for cells lacking LafA or LafK (**Figures 6H,I**). A strain lacking CheW showed an 80% reduction in swarming compared to wild-type, showing that the chemotactic system is required for proper swarming behavior. Interestingly, cells lacking ParC also showed a reduction in swarming colony formation of almost 50% compared to wild-type (**Figures 6H,I**). Thus, ParC plays a role in swarming of *V. parahaemolyticus*, suggesting that swarmer cells require a bi-polar localization of chemotactic signaling arrays in order to swarm in an optimal manner.

### Swarm-Colonies Consist of Different Cell Types with Distinct Localization Patterns of Chemotactic Signaling Arrays

**FIGURE 7:**
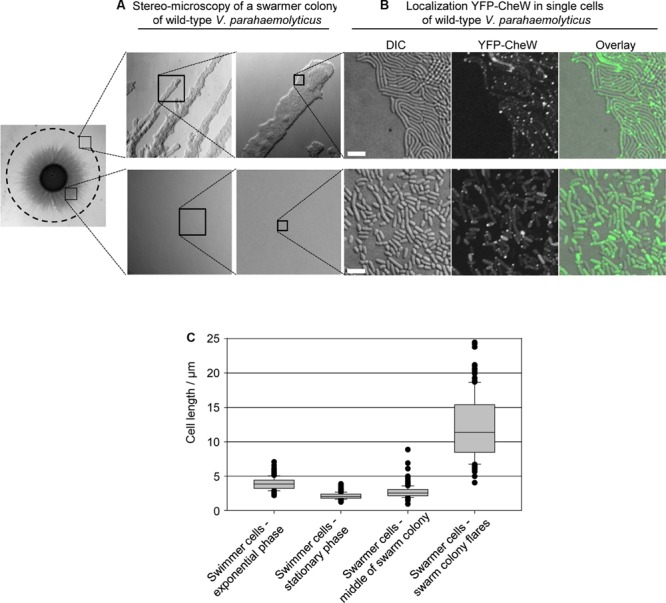
**Localization of signaling arrays in swarmer cells changes with swarm-colony development. (A)** Stereo-microscopy of a swarmer colony of *V. parahaemolyticus*. Upper panel shows the swarm flares at higher magnifications, and lower panel shows the middle of a swarm colony at higher magnifications. **(B)** Fluorescence microscopy showing the localization of YFP-CheW in *V. parahaemolyticus* cells from the periphery (swarm flares) and middle of swarm colonies. Images are representative of areas indicated in **(A)**. Cells from the periphery were imprinted on microscope slides. Due to cells being stacked in multilayers, cells from the middle were scraped off plates, re-suspended in medium and spotted on a microscope slide, in order to obtain single layered cells for microscopy. **(C)** Graph showing the cell-length distribution of *V. parahaemolyticus* depending on its growth phase and position within swarm colonies; swimmer cells – exponential phase, *n* = 297; swimmer cells stationary phase, *n* = 300; swarmer cell – middle, *n* = 307; swarmer cells – periphery, *n* = 151.

There are major differences in cell size – and likely also cell-type – according to the position of cells within a swarm colony [(Belas and Colwell, 1982), **Figure 7**]. In the periphery of the swarm colony, cells assemble into flares that extend outward from the colony. The flares consist of cells stacked in a few layers, thinning to a monolayer of long swarmer cells at the tip of the flares (**Figures 7A,C**). By contrast, closer to the center of the swarm colony cells are stacked in multiple layers and are considerably shorter than cells in the flares and more resembling stationary phase swimmer cells in cell length (**Figures 7A,C**). Due to the differences in internal organization of signaling arrays between swimmer and long swarmer cells, we hypothesized that *V. parahaemolyticus* also regulates its internal organization of signaling arrays depending on the cells position within swarm colonies. Thus, we analyzed the localization of YFP-CheW in cells originating from swarm flares and from the middle of the swarm colony (**Figure 7B**). In contrast to the long swarmer cells in the swarm-flares, in the vast majority of cells from the center of a swarm colony YFP-CheW localized to the cell poles in a uni- and bi-polar manner and only rarely was a lateral YFP-CheW cluster observed, just like seen in swimmer cells grown in liquid medium (**Figure 7B**). Thus, within swarm-colonies cells not only display morphological differences, but also regulate their intracellular organization and localization of chemotactic signaling arrays depending on their specific position within the colony.

## Discussion

We have performed a detailed analysis of the intracellular localization of chemotactic signaling arrays during the life cycles of *V. parahaemolyticus* and shown that in contrast to swimmer cells, where chemotactic signaling arrays are strictly confined to the cell poles, swarmer cells have two distinct localization patterns; bi-polar localization and lateral localization along the cell length (Gestwicki et al., 2000). Here, we show data suggesting that the formation and localization of polar and lateral arrays in swarmer cells are driven by distinct mechanisms. Additionally, we show that there is a correlation between swarmer cell length and the number of signaling arrays formed within the swarmer cell; long swarmer cells possess a higher number of signaling arrays compared to short swarmer cells and the number of lateral clusters formed increases with increased cell length. We show that lateral signaling arrays are localized in a non-regular manner but on a population average each cell halve hold the same amount of lateral signaling arrays. This distribution possibly ensures that upon cell division each daughter swarmer cell is likely to inherit laterally localized signaling arrays.

Using immunofluorescence on fixed cells it has previously been reported that lateral signaling arrays form in elongated cells of *V. parahaemolyticus* from liquid medium (Gestwicki et al., 2000). These experiments were performed in cells deleted for *lonS* that have partially initiated the swarmer cell differentiation program and are elongated in liquid media; the exact role of the protease LonS in regulation of differentiation, however, is not known, and pleiotropic effects cannot be excluded (Stewart et al., 1997). Here, we analyzed the localization in live genetically defined cells, both in *bona fide* swarmers and in artificially elongated swimmer cells from liquid medium that have not initiated the swarm program. We observe no formation of the non-regularly distributed arrays along the length of the artificially elongated swimmer cells, while the bona fide swarmers resemble cells lacking *lonS* in displaying lateral signaling arrays, hence suggesting that formation of non-regularly distributed signaling arrays is specific for cells that have initiated swarmer differentiation and not simply a consequence of cell elongation.

Interestingly, our data also suggest that swarmer cells employ two distinct mechanisms for localization of signaling arrays: First, the ParC system is responsible for recruitment of signaling arrays to the cell poles and the establishment of a bi-polar localization of signaling arrays. Second, a different mechanism regulates formation and positioning of lateral signaling arrays along the length of swarmer cells. Our data suggests that this mechanism specifically comes into action after entering the differentiation program to swarmer cells and is independent of cell length. It might be similar to the situation in the peritrichously flagellated *E. coli*, where formation of signaling arrays is driven by a stochastic process (Thiem and Sourjik, 2008), resulting in localization of signaling arrays at the cell poles and non-regularly along the cell length (Sourjik and Berg, 2000). *V. parahaemolyticus* encodes a large number of different chemoreceptors suggesting a pronounced capability for sensing and responding to environmental signals. Thus, one possibility is that one or more of these receptors are specific for swarmer cells and play a role in the formation of lateral signaling arrays: the ParC-system might not interact with those swarmer specific receptors, thereby allowing formation of lateral arrays driven by stochastic assembly similar to that observed for *E. coli* where individual receptors are inserted randomly in the membrane, in which they diffuse freely and either join existing arrays or nucleate new ones (Sourjik and Berg, 2000; Thiem and Sourjik, 2008). This suggestion is supported by microarray comparison between surface and liquid grown *V. parahaemolyticus*, where both up- and down-regulation in expression of several MCPs and predicted chemotaxis proteins, depending on the differentiation state, was detected (Gode-Potratz et al., 2011). Alternatively, the formation of lateral chemosensory arrays may be driven by a component of the lateral flagellar systems, which – similarly to the lateral chemotaxis arrays – are located in a non-regular manner along the length of the cell. It is likely that the formation and non-uniform distribution of signaling arrays along the cell length in swarmer cells ensures that chemotaxis sensory arrays are localized in close proximity to the randomly localized flagella along the cell body.

Interestingly, the actual role of chemotaxis during swarming is still not fully understood. It is known that in *E. coli* and *Salmonella* the chemotaxis system is required for swarming, however, there is also evidence indicating that it might not be chemotaxis *per se* that is required but instead the chemotaxis system plays a mechanical role in swarming motility (Burkart et al., 1998; Mariconda et al., 2006). Furthermore, it has been suggested that in *E. coli* interactions between cell bodies are responsible for swarm colony expansion and cell reversals rather than the chemotaxis system itself (Damton et al., 2010). In *V. parahaemolyticus* transposon insertion mutants have been isolated that simultaneously result both in chemotaxis and swarming defects, and experiments suggested that the chemotaxis system influences both the polar and lateral flagella systems (Sar et al., 1990; McCarter, 2004). The mutations map to two regions on chromosome 1 near the polar flagellar gene clusters, and likely insert in the chemotaxis gene operon. The exact insertion sites, however, have not been identified. Here, we used a clean genetic construct specifically deleted for the chemotaxis protein CheW, responsible for chemotactic behavior of swimmer cells (Ringgaard et al., 2014), and show that the swarming behavior of *V. parahaemolyticus* is clearly affected. Nonetheless, the actual mechanism by which chemotaxis influences swarming remains to be elucidated.

Our data also show that there are differences in the subcellular localization patterns of chemosensory arrays depending on the cell’s position within a swarm colony. It is possible that, as elongated swarmer cells spread over surfaces, cells that remain in the middle of the swarm colony dedifferentiate back into swimmers cells, and thus only position their signaling arrays at the cell pole in proximity of the polar flagellum required for swimming behavior. The natural habitat of *V. parahaemolyticus* is the marine environment, therefore it may be important to maintain a constant population of swimmers at all times. Thus, separating into two distinct cell populations within swarm colonies likely allows for swarming across surfaces while maintaining a continuous pool of swimmer cells that are ready to be released into liquid environments and immediately capable of exploring new surroundings.

## Author Contributions

JH carried out the experimental work, participated in data analysis, participated in the design of the study, and helped drafting the manuscript. SR masterminded the work, participated in data analysis, participated in the design of the study, and drafted the manuscript.

## Conflict of Interest Statement

The authors declare that the research was conducted in the absence of any commercial or financial relationships that could be construed as a potential conflict of interest.

## References

[B1] Alberti L. Harshey R. M. (1990). Differentiation of *Serratia marcescens* 274 into swimmer and swarmer cells. *J. Bacteriol.* 172 4322–4328. 2198253 10.1128/jb.172.8.4322-4328.1990 PMC213257

[B2] Alley M. R. Maddock J. R. Shapiro L. (1992). Polar localization of a bacterial chemoreceptor. *Genes Dev.* 6 825–836. 10.1101/gad.6.5.825 1577276

[B3] Bardy S. L. Maddock J. R. (2005). Polar localization of a soluble methyl-accepting protein of *Pseudomonas aeruginosa*. *J. Bacteriol.* 187 7840–7844. 10.1128/JB.187.22.7840 16267307 PMC1280319

[B4] Baumann P. Baumann L. (1977). Biology of the marine enterobacteria: genera beneckea and *Photobacterium*. *Annu. Rev. Microbiol.* 31 39–61. 10.1146/annurev.mi.31.100177.000351 334043

[B5] Belas M. R. Colwell R. R. (1982). Scanning electron microscope observation of the swarming phenomenon of *Vibrio parahaemolyticus*. *J. Bacteriol.* 150 956–959. 7068539 10.1128/jb.150.2.956-959.1982 PMC216449

[B6] Böttcher T. Elliott H. L. Clardy J. (2016). Dynamics of snake-like swarming behavior of *Vibrio alginolyticus*. *Biophys. J.* 110 981–992. 10.1016/j.bpj.2015.12.037 26910435 PMC4776037

[B7] Briegel A. Ortega D. R. Mann P. Kjær A. Ringgaard S. Jensen G. J. (2016). Chemotaxis cluster 1 proteins form cytoplasmic arrays in *Vibrio cholerae* and are stabilized by a double signaling domain receptor DosM. *Proc. Natl. Acad. Sci. U.S.A.* 113 10412–10417. 10.1073/pnas.1604693113 27573843 PMC5027440

[B8] Burkart M. Toguchi A. Harshey R. M. (1998). The chemotaxis system, but not chemotaxis, is essential for swarming motility in *Escherichia coli*. *Proc. Natl. Acad. Sci. U.S.A.* 95 2568–2573. 10.1073/pnas.95.5.2568 9482927 PMC19416

[B9] Damton N. C. Turner L. Rojevsky S. Berg H. C. (2010). Dynamics of bacterial swarming. *Biophys. J.* 98 2082–2090. 10.1016/j.bpj.2010.01.053 20483315 PMC2872219

[B10] Gestwicki J. E. Lamanna A. C. Harshey R. M. Mccarter L. L. Kiessling L. L. Adler J. (2000). Evolutionary conservation of methyl-accepting chemotaxis protein location in bacteria and archaea evolutionary conservation of methyl-accepting chemotaxis protein location in bacteria and archaea. *J. Bacteriol.* 182 6499–6502. 10.1128/JB.182.22.6499-6502.2000.Updated 11053396 PMC94798

[B11] Gode-Potratz C. J. Kustusch R. J. Breheny P. J. Weiss D. S. McCarter L. L. (2011). Surface sensing in *Vibrio parahaemolyticus* triggers a programme of gene expression that promotes colonization and virulence. *Mol. Microbiol.* 79 240–263. 10.1111/j.1365-2958.2010.07445.x 21166906 PMC3075615

[B12] Gode-Potratz C. J. McCarter L. L. (2011). Quorum sensing and silencing in *Vibrio parahaemolyticus*. *J. Bacteriol.* 193 4224–4237. 10.1128/JB.00432-11 21705592 PMC3147687

[B13] Harshey R. M. (1994). Bees aren’t the only ones: swarming in gram-negative bacteria. *Mol. Microbiol.* 13 389–394. 10.1111/j.1365-2958.1994.tb00433.x 7997156

[B14] Harshey R. M. Matsuyama T. (1994). Dimorphic transition in *Escherichia coli* and *Salmonella typhimurium*: surface-induced differentiation into hyperflagellate swarmer cells. *Proc. Natl. Acad. Sci. U.S.A.* 91 8631–8635. 10.1073/pnas.91.18.8631 8078935 PMC44660

[B15] Kirov S. M. Tassell B. C. Semmler A. B. T. Donovan L. A. O. Rabaan A. A. Shaw J. G. (2002). Lateral flagella and swarming motility in *Aeromonas* Species. *J. Bacteriol.* 184 547–555. 10.1128/JB.184.2.547 11751834 PMC139559

[B16] Maddock J. R. Shapiro L. (1993). Polar localization of the chemotreceptor complex in *Escherichia coli* cell. *Science* 259 1717–1723. 10.1126/science.8456299 8456299

[B17] Makino K. Oshima K. Kurokawa K. Yokoyama K. (2003). Genome sequence of *Vibrio parahaemolyticus*: a pathogenic mechanism distinct from that of *V. cholerae*. *Lancet* 361 743–749. 10.1016/S0140-6736(03)12659-1 12620739

[B18] Mariconda S. Wang Q. Harshey R. M. (2006). A mechanical role for the chemotaxis system in swarming motility. *Mol. Microbiol.* 60 1590–1602. 10.1111/j.1365-2958.2006.05208.x 16796690

[B19] McCarter L. (1999). The multiple identities of *Vibrio parahaemolyticus*. *J. Mol. Microbiol. Biotechnol.* 1 51–57. 10941784

[B20] McCarter L. Silverman M. (1990). Surface-induced swarmer cell differentiation of *Vibrio parahaemolyticus*. *Mol. Microbiol.* 4 1057–1062. 10.1111/j.1365-2958.1990.tb00678.x 2233248

[B21] McCarter L. L. (2004). Dual flagellar systems enable motility under different circumstances. *J. Mol. Microbiol. Biotechnol.* 7 18–29. 10.1159/000077866 15170400

[B22] McCarter L. L. (2010). Bacterial acrobatics on a surface: swirling packs, collisions, and reversals during swarming. *J. Bacteriol.* 192 3246–3248. 10.1128/JB.00434-10 20435735 PMC2897678

[B23] Milton D. L. O’Toole R. Horstedt P. Wolf-Watz H. (1996). Flagellin a is essential for the virulence of *Vibrio anguillarum*. *J. Bacteriol.* 178 1310–1319. 8631707 10.1128/jb.178.5.1310-1319.1996 PMC177804

[B24] R Development Core Team (2008). *R: A Language and Environment for Statistical Computing. R Found. Stat. Comput*. Vienna: R Foundation for Statistical Computing.

[B25] Rather P. N. (2005). Swarmer cell differentiation in *Proteus mirabilis*. *Environ. Microbiol.* 7 1065–1073. 10.1111/j.1462-2920.2005.00806.x 16011745

[B26] Ringgaard S. Hubbard T. Mandlik A. Davis B. M. Waldor M. K. (2015). RpoS and quorum sensing control expression and polar localization of *Vibrio cholerae* chemotaxis cluster III proteins in vitro and in vivo. *Mol. Microbiol.* 97 660–675. 10.1111/mmi.13053 25989366 PMC4646612

[B27] Ringgaard S. Schirner K. Davis B. M. Waldor M. K. (2011). A family of ParA-like ATPases promotes cell pole maturation by facilitating polar localization of chemotaxis proteins. *Genes Dev.* 25 1544–1555. 10.1101/gad.2061811.and 21764856 PMC3143943

[B28] Ringgaard S. Zepeda-Rivera M. Wu X. Schirner K. Davis B. M. Waldor M. K. (2014). ParP prevents dissociation of CheA from chemotactic signaling arrays and tethers them to a polar anchor. *Proc. Natl. Acad. Sci. U.S.A.* 111 E255–E264. 10.1073/pnas.1315722111 24379357 PMC3896188

[B29] Roth D. Finkelshtein A. Ingham C. Helman Y. Sirota-Madi A. Brodsky L. (2013). Identification and characterization of a highly motile and antibiotic refractory subpopulation involved in the expansion of swarming colonies of *Paenibacillus vortex*. *Environ. Microbiol.* 15 2532–2544. 10.1111/1462-2920.12160 23763278 PMC3908376

[B30] Sar N. McCarter L. Simon M. Silverman M. (1990). Chemotactic control of the two flagellar systems of *Vibrio parahaemolyticus*. *J. Bacteriol.* 172 334–341. 2294089 10.1128/jb.172.1.334-341.1990 PMC208437

[B31] Sourjik V. Armitage J. P. (2010). Spatial organization in bacterial chemotaxis. *EMBO J.* 29 2724–2733. 10.1038/emboj.2010.178 20717142 PMC2924652

[B32] Sourjik V. Berg H. C. (2000). Localization of components of the chemotaxis machinery of *Escherichia coli* using fluorescent protein fusions. *Mol. Microbiol.* 37 740–751. 10.1046/j.1365-2958.2000.02044.x 10972797

[B33] Stewart B. McCarter L. (2003). Lateral flagellar gene system of *Vibrio parahaemolyticus*. *J. Bacteriol.* 185 10.1128/JB.185.15.4508 PMC165745 12867460

[B34] Stewart B. J. Enos-Berlage J. L. McCarter L. L. (1997). The *lonS* gene regulates swarmer cell differentiation of *Vibrio parahaemolyticus*. *J. Bacteriol.* 179 107–114. 8981986 10.1128/jb.179.1.107-114.1997 PMC178667

[B35] Thiem S. Sourjik V. (2008). Stochastic assembly of chemoreceptor clusters in *Escherichia coli*. *Mol. Microbiol.* 68 1228–1236. 10.1111/j.1365-2958.2008.06227.x 18476921

[B36] Wadhams G. H. Armitage J. P. (2004). Making sense of it all: bacterial chemotaxis. *Nat. Rev. Mol. Cell Biol.* 5 1024–1037. 10.1038/nrm1524 15573139

[B37] Wadhams G. H. Warren A. V. Martin A. C. Armitage J. P. (2003). Targeting of two signal transduction pathways to different regions of the bacterial cell. *Mol. Microbiol.* 50 763–770. 10.1046/j.1365-2958.2003.03716.x 14617139

[B38] Wickham H. (2009). Hadley Wickham. *Media* 35:211 10.1007/978-0-387-98141-3

[B39] Yamaichi Y. Bruckner R. Ringgaard S. Cameron D. E. Briegel A. Jensen G. J. (2012). A multidomain hub anchors the chromosome segregation and chemotactic machinery to the bacterial pole. *Genes Dev.* 26 2348–2360. 10.1101/gad.199869.112 23070816 PMC3475806

